# A common-garden experiment to quantify evolutionary processes in copepods: the case of emamectin benzoate resistance in the parasitic sea louse *Lepeophtheirus salmonis*

**DOI:** 10.1186/1471-2148-14-108

**Published:** 2014-05-19

**Authors:** Lina Eva Robin Ljungfeldt, Per Gunnar Espedal, Frank Nilsen, Mette Skern-Mauritzen, Kevin Alan Glover

**Affiliations:** 1 Institute of Marine Research, P.O. Box 1870 Nordnes, N-5817 Bergen, Norway; 2 Sea Lice Research Centre, Department of Biology, University of Bergen, Box 7800, N-5020 Bergen, Norway

**Keywords:** *Lepeophtheirus salmonis salmonis*, Resistance development, Emamectin benzoate, Common-garden, Phenotypic variability, Parasite evolution

## Abstract

**Background:**

The development of pesticide resistance represents a global challenge to food production. Specifically for the Atlantic salmon aquaculture industry, parasitic sea lice and their developing resistance to delousing chemicals is challenging production. In this study, seventeen full sibling families, established from three strains of *Lepeophtheirus salmonis* displaying differing backgrounds in emamectin benzoate (EB) tolerance were produced and quantitatively compared under a common-garden experimental design. Lice surviving to the preadult stage were then exposed to EB and finally identified through the application of DNA parentage testing.

**Results:**

With the exception of two families (19 and 29%), survival from the infectious copepod to preadult stage was very similar among families (40-50%). In contrast, very large differences in survival following EB exposure were observed among the families (7.9-74%). Family survival post EB exposure was consistent with the EB tolerance characteristics of the strains from which they were established and no negative effect on infection success were detected in association with increased EB tolerance. Two of the lice families that displayed reduced sensitivity to EB were established from a commercial farm that had previously used this chemical. This demonstrates that resistant alleles were present on this farm even though the farm had not reported treatment failure.

**Conclusions:**

To our knowledge, this represents the first study where families of any multi-cellular parasite have been established and compared in performance under communal rearing conditions in a common-garden experiment. The system performed in a predictable manner and permitted, for the first time, elucidation of quantitative traits among sea lice families. While this experiment concentrated on, and provided a unique insight into EB sensitivity among lice families, the experimental design represents a novel methodology to experimentally address both resistance development and other evolutionary questions in parasitic copepods.

## Background

Aquaculture has become a major global industry. In Norway, the world’s largest producer of Atlantic salmon (*Salmo salar* L., 1758), annual production has grown from 98 tonnes in 1971 [1], to over 1.2 million tonnes in 2012 [2]. This rapid development has been met with a number of environmental challenges, for example interbreeding between farm escapees and wild conspecifics [3-5] and pathogen transmission [6]. Of the pathogens, the salmon louse (*Lepeophtheirus salmonis* Krøyer, 1837) (Crustacea: Copepoda: Caligidae), has emerged as one of the most critical economic [7,8] and fish-health related threats to the salmon farming industry [9,10]. Epizootics of *L. salmonis* on wild salmonids have been documented in fish farming intense areas [11-16] and have been linked with declines of wild salmonid populations in Europe [17,18] and North America [19,20].

*L. salmonis* is a naturally occurring marine ectoparasite of salmonid fishes in the northern hemisphere [21,22] and has recently been divided into two sub-species; *L. salmonis salmonis* occurring in the Atlantic, and *L. s. oncorhynchi* occurring in the Pacific [23]*L. s. salmonis* has coevolved with Atlantic salmonid fish hosts (*Salmo* spp.) [24] and has developed strategies required for survival, proliferation and host location in low densities across long distances [25]. The life cycle of *L. salmonis* comprises eight stages, each separated by moults [21,26,27]. The eggs hatch into the first of two non-feeding nauplii stages, followed by the infective copepodid stage. After locating and settling on a salmonid host, the louse develops through two filament-attached chalimus stages and two motile preadult stages into the final adult stage. The adult male fertilises the female immediately after her final moult. Throughout the rest of her life-time, the female protrudes up to 11 sets [28] of paired egg sacs (‘egg strings’) where 100–1 000’s of eggs [29] mature until they are released to hatch in the surrounding water masses.

A variety of methods for controlling *L. salmonis* on fish farms are employed or under development [10]. These include pest management strategies such as synchronised delousing [30], coordinated fallowing [31] and temporary protected zones [32,33]. They also include more direct control methods such as biological control with cleaner fish [34,35], selective breeding for resistant fish [36,37] and, potentially, vaccine development [38,39]. Nevertheless, despite the availability of a variety of methods, the industry is heavily reliant on anti-parasitic chemicals, applied as bath treatments or orally administered in-feed, to delouse fish in farms [40].

Reduced sensitivity of *L. salmonis* to the major chemical delousing treatments used in salmon farming was first observed in the early 1990´s when reduced effect of organophosphate treatments was documented [41,42]. More recently, reduced sensitivity or resistance to other delousing chemicals has also been documented, including hydrogen peroxide [43], pyrethroids [44,45] and the avermectin emamectin benzoate (EB: Slice®) [46-49]. In addition, instances of multiple resistance, i.e., reduced sensitivity or resistance to two or more chemicals at the same time, have recently been reported for *L. salmonis* in Norway [50]. Nevertheless, most of the actual mechanisms involved in resistance towards the main delousing chemicals used in salmonid aquaculture are at present unknown.

One of the major challenges to the further expansion of salmonid aquaculture is finding strategies to prevent, or at least delay [51], the development of pesticide resistance in sea lice. Bioassays used for testing the sensitivity of sea lice to different delousing chemicals have been developed and employed [52,53] as part of resistance management strategies in countries where resistance or decreased sensitivity has been reported. These bioassays give average sensitivity values for populations or groups of tested individuals, such as the effective concentration (EC_50_), which is defined as the concentration of a compound that immobilise 50% of the target organism (moribund + dead) [54]. Bioassays for given delousing compounds are increasingly used prior to treatment on commercial salmon farms, in order to examine the sensitivity level of sea lice present on the fish. The likely outcome of a treatment is then identified by comparing the EC_50_ to the therapeutic concentration achieved in the fish as a result of treatment by that compound. Importantly however, these bioassays do not accurately quantify how a potential reduced sensitivity is distributed within the population being tested. For example, does a 30% higher EC_50_ value at one farm imply that all lice are approximately 30% less susceptible than a baseline population, or are 30% of the lice completely resistant? This question is important in order to help identify the underlying mechanisms of resistance, their distribution in the population, and the likely evolutionary consequences of treatment.

Common-garden experiments involve the comparison of genetically distinct strains, families or populations under identical environmental conditions. Such experimental protocols are often used to disentangle the effects of genetic and environmental variation on the phenotype. Within fish, common-garden experiments have been widely used to identify genetic differences among populations, and are often accompanied with DNA parentage testing in order to identify offspring to their genetic group of origin that have been communally reared in artificial [55-57] as well as natural habitats [58-60]. However, for parasites which require access to a host, common-garden experiments are very rare, and none have been described for either parasitic or non-parasitic copepods. Thus, establishing a common-garden experimental system for *L. salmonis* would be highly valuable for investigating the development of resistance in *L. salmonis*, and specifically, to quantify susceptibility differences among families as well as strains. In addition, such a system would provide a powerful tool to address the evolution of other traits in this parasite such as fecundity and virulence [61].

The present study had two primary aims: 1. To establish a common-garden experimental system for testing variation in phenotypic traits between full sibling families of lice reared in a communal environment, and 2. To test the performance of the experimental system by looking at the distribution of decreased sensitivity among full sibling families of *L. salmonis* to emamectin benzoate (EB) which is the active ingredient in Slice® and the most commonly used oral medicine against sea lice [40] since its introduction in 1999.

## Methods

### Overall experimental design

**Figure 1:**
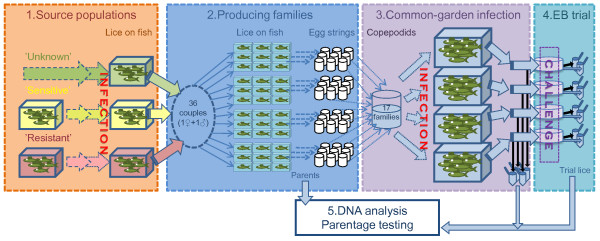
**Overview of the experimental design.** The five step experimental procedure: 1. Synchronized single-strain parent populations of *L. salmonis* are produced from three source populations of different origin. 2. Couples of virgin lice in individual fish-tanks produce full sibling families 3. A mixture of copepodids from selected louse families common-garden infected into four replicate tanks. 4. Sampled lice exposed to EB, evaluated and sorted. 5. Family affiliations are resolved by individual genotyping of offspring, sorted by trial outcome, and parents. Full arrows: Preadult II female and adult male lice, in pairs of two () or a group of many () lice. Full black arrows () denotes dead lice. Dashed arrows: Egg strings from one () or many () female lice. Dotted arrows: Copepodids originating from one () or many () female lice.

In order to establish an experimental framework in which to investigate variability in phenotypic traits within and among salmon lice populations, an experimental protocol was established for producing full sibling family groups to study in a common-garden experiment (i.e., mixing all families together in the same environment) (Figure 1). The protocol, which in this experiment was designed to address variation in susceptibility to emamectin benzoate (EB), consists of a sequence of five experimental parts, summarised as follows: 1. Producing single-strain parental populations of *L. salmonis* that are synchronised in developmental timing, 2. Creating full sibling families by keeping couples of virgin lice separated in individual fish tanks, 3. Common-garden infection in replicate multiple-fish tanks with a mixture of copepodids from a number of selected families, 4. Sampling all individuals that had survived from the infections, for sensitivity testing in a post-termination trial, here: for susceptibility to EB. 5. Genotyping all parents and offspring in order to identify individuals back to family of origin, and thus examine family and strain based differences in performance. Specific details for each of the experimental components described above are given in the sections ‘Production of parents and experimental families’, ‘Common-garden infection’, ‘Emamectin benzoate trial’ and ‘Genotyping and parentage testing’ below.

### Animal welfare considerations and rearing conditions

Although salmon lice belong to the systematic entities that are not protected by animal welfare legislation, the development of *L. salmonis* past the infective copepodid stage requires the attachment to a host fish. The Norwegian Animal Welfare Act strictly regulates the maintenance of fish used as hosts for salmon lice. All parts of this study were conducted in accordance with these regulations, under the application 2009/186329, in the wet laboratory of the Institute of Marine Research (IMR) in Bergen. Here, farmed Atlantic salmon (*Salmo salar*) in the size range of ca 200 to 700 g were used as host for cultivation of salmon lice and experiments involving salmon louse infections.

The fish used for culturing the lice strains were kept in 250 L and 500 L multi-fish tanks. These were hand fed once daily on a commercial diet. Tank water exchange, aeration and current was regulated by inlet water flow kept at ca 60 L∙hr^-1^ per kg fish (minimum 400 L∙hr^1^, in order to maintain sufficient flow in the tank), of natural seawater pumped up from 120 m depth and passed through a column aerator. The water temperature kept 9.0 ± 1°C and salinity 34 ± 0.5‰ throughout the year. The tanks were kept indoors at an artificial, 12 hrs daily (fluorescent) light regime under transparent lids.

The fish used as hosts during the production of full sibling salmon louse families, by pairing a single male and a virgin female louse together on a single fish, were placed individually in an array of 36 plastic tanks, each of 50 L volume. This setup was similar to that described by Hamre and Nilsen [62], and are hereafter referred to as the single-fish tanks. All procedures involved in propagating, handling and quantification of *L. salmonis* were performed using the methods and culturing systems described in detail by Hamre et al. [63].

### Source lice populations

Pesticide resistance is generally defined as the inherited ability in a strain of pest to tolerate doses of toxicants that would prove lethal to the majority of individuals in a sensitive population of the same species [64]. However, there are a multitude of context-specific definitions for resistance [65,66], some of which include whether the population exceeds a specific threshold ratio to a known sensitive strain. In the present study, three source populations (strains) of *L. salmonis*, with different histories of EB exposure, were used to produce the experimental families. These strains are here-on referred to as ‘resistant’, ‘sensitive’ and ‘unknown’. This classification was chosen in order to aid presentation and enable consistency throughout the paper, despite the fact that the level of sensitivity for each of these strains had not been accurately quantified prior to initiating the experiment.

All strains of lice used in this study originated from the Atlantic, and belong therefore to the sub-species *L. s. salmonis*[23]. The ‘resistant’ strain was represented by *L. salmonis* collected by the Norwegian Food Authority (Mattilsynet) in September 2008 at a salmon farm in Austevoll on the west coast of Norway. This farm had reported multiple treatment failures with Slice®. After collection on the farm, >120 egg string pairs were transported to the wet laboratory facilities at IMR. The strain had been cultivated for six generations prior to being used in the present study (without additional exposure to EB), and sub-sets of this strain have displayed reduced sensitivity to EB in tests performed on previous generations [48].

The ‘sensitive’ strain used in the present study consisted of the third generation of an *L. salmonis* strain originating from 15 fertilised females collected from wild sea trout, *Salmo trutta* L., in Oslofjord, eastern Norway, in October 2009. The strain was assumed to be susceptible to EB, thus denominated ‘sensitive’, based on the fact that there is no commercial farming of salmon in this region, and therefore, salmon lice in this region have not been recently exposed to EB.

The third experimental strain included in this study was denominated ‘unknown’ with respect to its susceptibility to EB. This strain was founded by 35 fertilised salmon louse females collected from a salmon farm located on the island of Strøno, Hordaland, west of Norway on 16 April 2010. The farm had not previously reported treatment failure with Slice®. However, this farm is located in a dense farming region where other farms, such as the one located in Austevoll, had experienced previous problems of reduced efficacy of EB. After collection, the females were transported to the laboratory in ambient seawater from the farm, transferred onto previously uninfected Atlantic salmon within three hours and were left undisturbed for one egg string cycle (ten days) before the new egg strings were collected.

### Production of parents and experimental lice families

The production of synchronised parental strains was initiated on 26 April 2010. Pairs of egg strings were collected and incubated from 15 females from six fish each of the ‘sensitive’ and the ‘unknown’ strains, and from 37 ‘resistant’ strain females from 14 fish. The egg strings were maintained in three 2 L flow-through incubators [for a detailed description, see: [63], each representing one strain. Fourteen days later, the resulting three batches of infective copepodids were used to separately infect three multi-fish tanks, each containing 15 previously uninfected Atlantic salmon. Thus, at this stage, the strains were still maintained separately (Figure 1).

Polyandry has been previously documented in *L. salmonis*[67]. Therefore, in order to produce full sibling families where control over both paternal and maternal contribution was maintained, and in order to produce hybrids between strains, virgin female lice were collected from each of the experimental strains. These were collected as preadult II females, 35 days post infection (DPI), to ensure that they had not been fertilised (which occurs in association with the moult from preadult II to adult [68]). At this stage the majority of the males were adults, ready to fertilise females upon moulting. From the collected lice, a single female and male couple were placed onto one salmon, each maintained individually in single-fish tanks, in order to ensure single paternity. A total of 36 couples were established as follows; ‘Resistant’ (N = 6), ‘Sensitive’ (N = 6), ‘Unknown’ (N = 12), and the ‘Hybrid’ (N = 12) groups. Hybrid couples were produced by pairing a sensitive male with a resistant female (SxR, N = 6), or a resistant male with a sensitive female (RxS, N = 6).

Each of the 36 single-fish tanks contained a filter to capture lice that detached from their hosts. This was checked for lice twice daily in connection with hand feeding of the fish. Living lice encountered in filters were reattached to the fish manually using forceps. Lice found dead in the filter, or alive but unable to reattach to its host upon multiple attempts, were removed from the experiment and preserved individually on 99.5% ethanol.

The first pair of egg strings extruded by a female *L. salmonis* after fertilization are always shorter [28] and may exhibit more variable hatching success (pers. obs.) than all subsequent pairs. On 66 DPI; the majority of the females had protruded their second set of egg strings. On this day, all egg strings were collected from female lice (N = 33) still attached to their host fish. The collected egg strings, 29 pairs and three single strings (one female carried no egg strings) (Table 1), were incubated individually in the system for small incubators described by [63]. The 33 females, and the male lice associated with them, were preserved on 99.5% ethanol in individually marked 2 ml tubes, and stored along with those that had detached from the fish previously.

**Table 1:** **Production of *L. salmonis *** **families from different experimental groups**

**Group**	**Couples**	**Egg strings**	**No hatching**	**Hatchlings excluded**	**No male**	**Families produced**
Resistant	6	5*	1	2	0	2
Sensitive	6	5**	1	2	1	1
Hybr (SxR)	6	5	0	1	0	4
Hybr (RxS)	6	5	0	1	1	3
Unknown	12	12	3	2	0	7
Total	36	32	5	8	2	17

Group = experimental group, defined by the strain(s) of the mother and the father, where Resistant = both parents resistant, Sensitive = both parents sensitive, Hybr = hybrids (SxR: sensitive father and resistant mother; RxS: resistant father and sensitive mother) and Unknown = both parents unknown. Couples = initial number of *L. salmonis* couples from each group; maximum number of potential families. Egg strings = number of egg string pairs incubated from each group, differ from ‘couples’ if the female was lost during the experiment or did not carry egg strings. No hatching = number of egg string pairs that did not hatch within 11 days in incubation. Hatchlings excluded = number of groups of hatchlings containing few, or inactive, copepodids (rejected from further use in the experiment). No male = number of families excluded due to loss of the male in the single-tank. Families produced = number of families meeting the requirements for further use in the experiment. Single egg strings were also collected and incubated. *: one female carried only one single egg string. **: two females carried only one single egg strain each.

### Common-garden experiment

Fourteen days after incubation of the egg strings from single families, the hatching success for each family was evaluated by visual inspection as: ‘Hatched’: living copepodids observed, or ‘Not hatched’: no living copepodids and/or unhatched egg strings observed in the incubator. Families of reduced viability (containing few copepodids, a large fraction of dead nauplii or copepodids, or notably low activity among the hatchlings compared to other families) were rejected from further use. Families resulting from successfully hatched second egg string pairs, for which both parents were available for subsequent DNA parentage analysis, were used in the common-garden experiment (Table 1). Based on these criteria, 17 families were selected and denoted ‘Family 1’ to ‘Family 17’ (Table 2). All copepodids from each of the selected families were counted in a counting chamber with the assistance of a stereo microscope. They were thereafter mixed together into a single glass beaker containing seawater from the hatchery’s water inlet. The mixture, containing a total of 4 554 copepodids from 17 families, was carefully stirred to ensure a random and even distribution. Thereafter, this was split in four equal parts, and used to infect four replicate 500 L tanks, each containing 20 Atlantic salmon that had not previously been exposed to sea lice. Lice development and host fish status were visually inspected daily. In order to compare individuals of equal size and age, bioassay tests of tolerance to chemicals are primarily carried out on preadult lice. By experimental design, our trial lice were already synchronised in age, therefore, sampling for the EB challenge was scheduled for the last point in time in the development when lice of both genders are equally sized. This occurs when males are adult, and females are still at the second preadult stage (which occurs in the same time span since the males’ rate of development is faster than the females’).

**Table 2:** **Summary of background information and trial results for the 17 families of *L. salmonis***

**Family**	**Background**	**Common-garden infection**				**EB challenge**		
**Nr**	**Group**	**N**_**0 **_**(cops)**	**n S**_**CG**_	**% S**_**CG**_	**Tank distr %**	**N**_**0 **_**(lice)**	**n S**_**EB**_	**% S**_**EB**_
1	Resistant	278	124	44.6	23 - 27	123	57	46.3
2	Resistant	214	97	45.3	23 - 32	95	54	56.8
3	Hybr (SxR)	270	117	43.3	21 - 33	115	73	63.5
4	Hybr (SxR)	276	128	46.4	17 - 34	123	91	74.0
5	Hybr (SxR)	91	41	45.1	15 - 32	41	16	39.0
6	Hybr (SxR)	191	79	41.4	22 - 33	78	32	41.0
7	Hybr (RxS)	181	81	44.8	20 - 31	81	24	29.6
8	Hybr (RxS)	582	288	49.5	23 - 27	285	117	41.1
9	Hybr (RxS)	384	192	50.0	19 - 30	190	133	70.0
10	Sensitive	171	49	28.7	22 - 33	49	4	8.2
11	Unknown	325	151	46.5	22 - 29	149	14	9.4
12	Unknown	450	195	43.3	21 - 30	190	15	7.9
13	Unknown	318	60	18.9	20 - 30	57	5	8.8
14	Unknown	230	91	39.6	22 - 29	91	13	8.8
15	Unknown	157	77	49.0	21 - 29	77	29	37.7
16	Unknown	240	103	42.9	20 - 33	101	9	8.9
17	Unknown	196	93	47.4	23 - 28	90	27	30.0
All		4 554	1 966	43.2	21 - 30	1 935	713	36.8

Group = experimental group, defined by the strain(s) of the mother and the father, see Table 1. N_0_ (cops) and N_0_ (lice) = initial numbers of copepodids and lice. n S = number of survivors at termination of CG = common-garden infection, and EB = emamectin benzoate trial, respectively. Tank distr % = between-tank distribution of individuals from the family at termination time, given as the minimum - maximum values from the four tanks. % S = number of surviving individuals as fractions of the initial numbers, in CG and EB, as described above.

The common-garden experiment was terminated on 1 September 2010, at 316 degree days post-infection (DDPI) (34 DPI at a mean temperature of 9.3°C). One at a time, all fish were anaesthetised for 2 to 3 minutes in metomidate (5 mg∙l^-1^) and benzocaine (60 mg∙l^-1^) in seawater. The lice were carefully removed from the fish by forceps and placed in four 5 L glass beakers containing seawater, one beaker per tank. The beakers were partially immersed in running water, in order to keep the temperature stable. Lice that had fallen off in the anaesthetic bath or died during sampling (e.g., due to forceps treatment), were preserved on 99.5% ethanol in four tubes, each representing one tank replicate. The surviving lice, located in the four glass beakers, were subsequently exposed to a one-dose EB trial.

### Emamectin benzoate trial

The one-dose sensitivity trial with EB was performed using the bioassay methodology detailed in [54]. A work solution (1 ppm EB) was prepared by diluting 10 ml of 100 ppm stock solution (EB in methanol) with 990 ml of seawater. EB exposure was started by adding 225 ml of the work solution to each of the four glass beakers that contained 4 225 ml of seawater and lice from the replicate tanks. This resulted in a 50 ppb EB concentration. Beakers containing lice were thereafter incubated overnight at 9.2°C ± 0.2°C. After 20 hours exposure, all lice were evaluated and sorted as survivors (living) vs. immobilised (moribund + dead) and preserved on 99.5% ethanol for storage and subsequent parentage analysis. This evaluation was conducted in accordance with the response criteria detailed in the ‘Handbook in resistance management’ [54], as follows: *Living:* Attached to the wall of the beaker or actively swimming behaviour. *Moribund:* Not capable of attaching to a surface (the inside wall of the beaker), using the flat body as a ‘sucking disc’. Movements of extremities or internal organs could still be observed. *Dead:* No movements, neither extremities nor gut or other organs. Due to the large number of lice that needed to be quickly classified, no distinction was made between moribund and dead lice.

Evaluation of the lice, starting with those from the ‘Tank 1’ beaker, took approximately one hour for each beaker. Consequently, the duration of EB exposure was 20, 21, 22 and 23 hours in the replicate beakers from tanks 1, 2, 3 and 4 respectively. In order to test if the exposure time differences affected the results, an exposure time corrected dataset to be tested in parallel with the actual survival data was established. This corrected data set was obtained for each tank from the ratio ‘% dead lice divided by exposure time’ multiplied by the average exposure time for all tanks, assuming (which is not necessarily correct) a negative linear relationship between survival and EB exposure time.

### Genotyping and parentage testing

All offspring sampled in this study were identified back to their family of origin by screening highly polymorphic microsatellite loci and matching their multi-locus genotypic profiles to pairs of parents using the genotype-exclusion based family assignment program FAP v. 3.6 [69].

DNA from all parents and offspring were extracted in a 96-well format using the commercially available Qiagen DNeasy kit. A minimum of 2 blank controls per tray were used. All of the samples were analysed with the following five microsatellite loci amplified in a single PCR reaction. Multiplex 1 = *LsalSTA1*, *LsalSTA2*, *LsalSTA4*, *LsalSTA5*[70] and *LsNUIG14* adapted by Todd et al. [70]. A low number of individuals displayed multiple-locus genotypes that matched between families with these five loci. For these individuals, including their parents, it was necessary to analyse the following loci in another two multiplex reactions in order to conclusively resolve their family of origin. Multiplex 2 = *Lsal103EUVC*, *Lsal109EUVC*, *Lsal110EUVC*, *Lsal111EUVC*[71] and *LsNUIG09*[72]. Multiplex 3 = *Lsal104EUVC*, *Lsal105EUVC*, *Lsal106EUVC*, *Lsal108EUVC*[71], *LsalSTA3*[70] and *LsNUIG35B*[73]. Amplification conditions are available from the authors, upon request. All of these loci have been previously used in this laboratory to examine population genetic structure of lice throughout the Atlantic (Glover et al., 2011). PCR fragments were separated on an ABI 3730XL sequencer and sized relative to the GeneScan™ 500LIZ™ size standard by Applied Biosystems. Alleles were scored twice by independent observers, following automatic binning implemented in the Genemapper (v. 4.0) software.

### Data analyses

#### Common-garden infection

In order to test for any potential tank-effects, a chi-square test for goodness-of-fit was applied to the total number of individuals collected from the four tanks on termination day. A further three chi-square tests for independence between tanks were applied to the number of individuals from different families, experimental groups and gender, separately.

Generalized linear mixed models were applied to test the effects of family, parental strain and experimental group on infection success. There were two parental strain variables: ‘sire’ (strain of the father; sensitive, resistant or unknown) and ‘dam’ (strain of the mother; sensitive, resistant or unknown). The ‘experimental group’ variable was tested twice; as ‘group (1H)’ where the seven hybrid families were pooled into one group, and as ‘group (2H)’ where the hybrid families were separated into two groups; ‘RxS’ and ‘SxR’. The groups ‘Resistant’, ‘Sensitive’ and ‘Unknown’ were included in both of these alternatives. Infection success was entered as a binary response and groups and parental strain was entered as fixed effects in the models, assuming binomially distributed error. Family was entered as a random effect to account for dependency between individuals within families [74].

Due to the limited number of individuals in some combinations of levels of these variables, the effects of groups and parents were tested in separate models. The effects of these variables were therefore assessed in an *a priori* formulated set of competing models (Table 3) following the information-theoretic approach [75]. Assessing the explanatory power of family as a random factor is complicated in mixed effects logistic regressions, as estimation of intra-family correlation is not clear-cut [76]. Therefore, we also included a generalized linear model with family as a fixed variable only. The models were ranked (Table 3) and the best model selected using Akaike’s information criterion (AIC) [75].

**Table 3:** Models applied to infection success in the common-garden experiment, ranked by AIC

**Model**	**Fixed effect**	**Random effect**	**df**	**AIC**	**ΔAIC**
Cops.m5	Family	-	17	6 136.0	0
Cops.m0	-	Family	2	6 157.4	21.4
Cops.m1	Group (1H)	Family	5	6 158.1	22.1
Cops.m4	Sire	Family	4	6 159.2	23.2
Cops.m2	Group (2H)	Family	6	6 159.5	23.5
Cops.m3	Dam	Family	4	6 160.6	24.6
Cops.m0B	-	-	1	6 229.0	93.0

A set of generalized linear models (GLM) and generalized linear mixed effect models (GLMM) testing for the relative importance of different background covariates on the infection success. GLMs include fixed effect variables, and GLMMs contain both fixed and random effects. Response variable: Infection success status (binary) from common-garden trial, where unsuccessful individuals were lost somewhere between infection and sampling and all individuals sampled on termination day were defined as successful.

Covariates (fixed and/or random effect variables): Family = Families nr 1–17.

Group (1H) = Resistant, Sensitive, Hybrid, Unknown.

Group (2H) = Resistant, Sensitive, Hybrid SxR, Hybrid RxS, Unknown.

Sire (source strain of the father) = Resistant, Sensitive, Unknown.

Dam (source strain of the mother) = Resistant, Sensitive, Unknown.

Test results: degrees of freedom (df), AIC values and changes in AIC (ΔAIC).

Due to large differences in numbers of copepodids produced per family, the potential relationship between number of copepodids produced by one family, and that specific family’s infection success was investigated by logistic regression. Family 2 (emerging from one single egg string) and Family 5 (from a short first set pair of egg strings) were excluded from this analysis, due to their deviating egg string properties.

#### Emamectin benzoate challenge

A chi-square test for goodness-of-fit was applied to test for tank effects on total survival (with and without correction for exposure time) in the EB trial. Chi-square tests for independence between tanks were then applied to the survival (with and without correction for exposure time) of different families, experimental groups and gender, in separate tests.

Another set of generalized linear mixed models were formulated in order to compare the different covariates’ influence on the EB trial results.

It is possible that the parental contribution to EB susceptibility differ in hybrids depending whether the mother or the father is the less sensitive parent. Therefore, the results were tested for the two alternative versions of the ‘experimental group’ variable: ‘group (1H)’ and ‘group (2H)’, to see if the hybrids should be considered as one or two groups in the analysis.

A logistic regression mixed model was fitted to test which factors influenced survival of the EB treatment, with exposure time, gender of individuals and experimental group as fixed variables and family as a random factor. The covariates exposure time and tank were confounded since the exposure time had differed for samples from different tanks. The exposure time corrected data set, while useful for comparing group level results expressed as ratios (i.e. infection success or EB survival as fractions of the family or group totals), could not be applied on the individual level, given the binary nature of the survival data. To assess potential tank effects redundant of exposure time, the Pearson residuals of the model were plotted against tanks and no such effects were observed (Additional file 1: Figure S1). Thus, we conclude that the covariate ‘tank’ was not likely to independently contribute to the survival frequency in the EB challenge. Again, a full model including all explanatory variables could not be fitted, due to the limited number of individuals in some combinations of levels of these variables, and the fact that some of the variables were confounded. We therefore formulated an *a priori* set of models and identified the best model based on AIC (Table 4). The models were formulated with the individuals’ post-trial status (survivor/immobilised) as the binary response, family as random intercept, and the rest of the explanatory variables: experimental group, gender, sire, dam and exposure time, as fixed effects (Table 4). Exposure time was entered as a numeric covariate. Family was entered as a random effect to account for autocorrelation within families when assessing the relative importance of other explanatory variables. As stated for the common-garden analysis above, we also included models where family was defined as a fixed effect, in order to assess the explanatory power of family.

**Table 4:** Models applied to survival data from the emamectin benzoate challenge, ranked by AIC

**Model**	**Fixed effects**	**Random effects**	**df**	**AIC**	**ΔAIC**
eb.m13	Family, Exposure time, Gender	-	19	2 084.7	0
eb.m12	Family, Exposure time	-	18	2 099.3	14.6
eb.m11	Family	-	17	2 109.4	24.7
eb.m1	Exposure time, Gender, Group (1xH)	Family	7	2 111.9	27.2
eb.m2	Exposure time, Gender, Group (2xH)	Family	8	2 113.4	28.7
eb.m4	Exposure time, Gender, Dam	Family	6	2 115.5	30.8
eb.m3	Exposure time, Gender, Sire	Family	6	2 117.6	32.9
eb.m5	Exposure time, Gender	Family	4	2 125.0	40.3
eb.m6	Exposure time, Group (1xH)	Family	6	2 126.0	41.3
eb.m7	Exposure time, Group (2xH)	Family	7	2 127.5	42.8
eb.m9	Exposure time, Dam	Family	5	2 130.1	45.4
eb.m8	Exposure time, Sire	Family	5	2 132.2	47.5
eb.m10	Exposure time	Family	3	2 139.5	54.8
eb.m14	Exposure time	-	2	2 537.9	453.2

GLMs and GLMMs testing for the relative influence of different background covariates on the EB trial results. The models are ranked based on AIC, where a lower AIC value reflects a better model fit to the data.

Response variable: Status after the EB challenge; living or immobilised/dead.

Covariates (fixed and/or random effect variables): Family, Group (1H), Group (2H), Sire and Dam = as listed for Table 3. Exposure time = 20, 21, 22 or 23 hours of EB exposure. Gender = female or male.

Test results: degrees of freedom (df), AIC values and changes in AIC (ΔAIC).

Potential relationship between clutch size and the families’ response in the EB trial was investigated by a separate logistic regression. Family 2 and Family 5 were excluded from this analysis as for the infection success.

All statistical analyses were conducted using the computing environment R [77] with mixed-effects models fit using *lme4*[78]. In all significance tests, the tests were considered significant at the *P* < 0.05 level.

## Results

### Production of experimental families in single-fish tanks

During the stage of family production when louse couples were kept in single-fish tanks (31 days from couple formation to sampling), two lice, one of each sex, were found alive in the filters and were successfully reattached to the fish. Five female and eight male lice permanently detached from their host fish. Out of these, seven were recovered from the filters and sampled onto 99.5% ethanol tubes. However, a total of three females (one from the resistant, and two from the sensitive strains) and three males (two from the resistant, and one from the sensitive strains) could not be accounted for. These lice were possibly consumed by the fish.

Due to loss of lice parent(s), absence of egg strings, egg strings hatching to a low degree or not at all or inactive copepodids, 19 couples out of the initial 36 were excluded from further use in the experiment. See Table 1 for a summary of the rejection statistics. Among the 17 families selected for the remainder of the experiment, seven originated from the unknown strain, two families represented the resistant strain, and seven families were hybrids (resistant sire × sensitive dam (RxS) N = 3, and sensitive sire × resistant dam (SxR) N = 4). Only one family from the sensitive laboratory strain could be used, primarily due to low hatching results.

In 15 of the selected families, the offspring originated from a full pair, i.e. two strings of 2^nd^ sets of egg strings. Family 2 (N = 214 copepodids) hatched from one single egg string and Family 5 (N = 91 copepodids) hatched from a short 1^st^ set pair of egg strings. The numbers of copepodids contributed by the 17 families to the infection batch (268 ± 119; mean ± SD) varied from 157 (Family 15) to 582 (Family 8).

### Common-garden experiment

In this study, the term ‘infection success’ has been chosen to represent the relationship between the number of lice recovered from the tank at 34 DPI, as a percentage of the number of copepodids used in the infection. Of the total 4 554 copepodids used to infect the four replicate tanks, 1 966 (492.5 ± 33; mean ± SD for tanks) managed to successfully attach to fish and survive until the termination on 34 DPI. This gives an overall infection success of 43%.

There were no significant differences (Goodness of fit: χ^2^ = 6.71, 3 df, *P* = 0.082) in overall infection success between the four tanks. The overall proportion of females and males in the total population was 52 and 48%, and there was no significant difference in the gender frequency between the four replicates (χ^2^ = 3.35, 3 df, *P* = 0.34). The vast majority, i.e., > 98% of the sampled lice, were preadult II females and adult males.

In order to identify the 1 966 salmon lice recovered from the four tanks to experimental group and family of origin, these individuals were subject to parentage testing using microsatellite loci. After genotyping with the first multiplex combination of five loci the family affiliations were established for a total of 1, 910 individuals (97%). A second round of analysis for 11 loci, resolved the pedigree of the remaining (n = 56) individuals, giving 100% family assignment for the data set.

**Figure 2:**
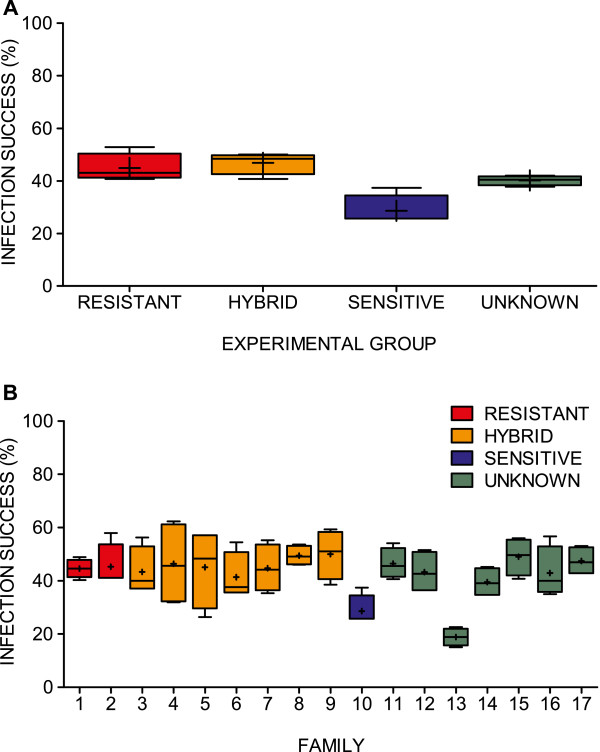
**Infection success variability in the common-garden experiment.** Ratios (%) between the numbers of *L. salmonis* individuals sampled at termination day and the initial numbers of copepodids at infection, when separated by the four experimental groups **(A)**, and by the 17 families **(B)**. Data presented from the four replicate tanks, where coloured boxes represents the 25-75% quartile, whiskers at min - max, line at median and (+) at mean values.

Given the pedigree of all individuals it could be concluded that there were no tank effects in the number of surviving lice per family (χ^2^ = 42.6, 48 df, *P* = 0.69), or experimental group (χ^2^ = 3.88, 9 df, *P* = 0.92), in the four tank replicates on termination day. Thus, infection success data for groups and families was pooled over the four replicate tanks (Figure 2A and B). Using these pooled data, the infection success ranged from 19% to 50% between the families (χ^2^ = 116.7, 16 df, *P* < 0.0001) (Figure 2B). Despite differences in the initial numbers of copepodids used per family (see Table 2), the frequency of surviving individuals was consistent among groups and families, with two exceptions that displayed markedly lower infection success: the ‘Sensitive’ Family 10 (29%), and Family 13 (19%) from the ‘Unknown’ group. If these two families were excluded from the dataset, there were no significant difference in infection success between the remaining 15 families (χ^2^ = 14.8, 14 df, *P* = 0.39), suggesting that the low infection success of these two families accounted for the observed difference in infection success between families.

As judged by the AIC, the best model for explaining the infection success was model Cops.m5 (Table 3), including family only, as a fixed variable. The variable was highly significant (Anova, analysis of deviance: df = 16, *P* < 0.001), and a dispersion factor of 1.004 indicated a good model fit. However, the model only explained 2% of the total deviance in the data. The low difference between AIC values between models Cops.m0 to Cops.m4 indicates that the influences of experimental group and parental strain were of minor importance when family was included as a random factor. However, as previously noted, infection success for Family 10 and Family 13 deviated from the rest of the families. In order to investigate how and if these two families influenced the total family effect on the infection success, the same set of models was run for a data set where these two families were excluded. This time, the model where family was included as fixed effect was ranked last (Additional file 2: Table S1), suggesting that the previously identified family effect on the infection success could be attributed to these two families primarily.

No relationship was detected between a family’s initial number of copepodids and infection success (R^2^ = 0.036, *P* = 0.50), indicating that, in this case, infection success was independent of clutch size upon initiation of the experiment.

### Emamectin benzoate (EB) trial

Less than 2% (31 of 1 966) of the salmon lice died prior to the EB trial, as a result of handling during sampling. These individuals were not included in the one-dose EB exposure.

**Figure 3:**
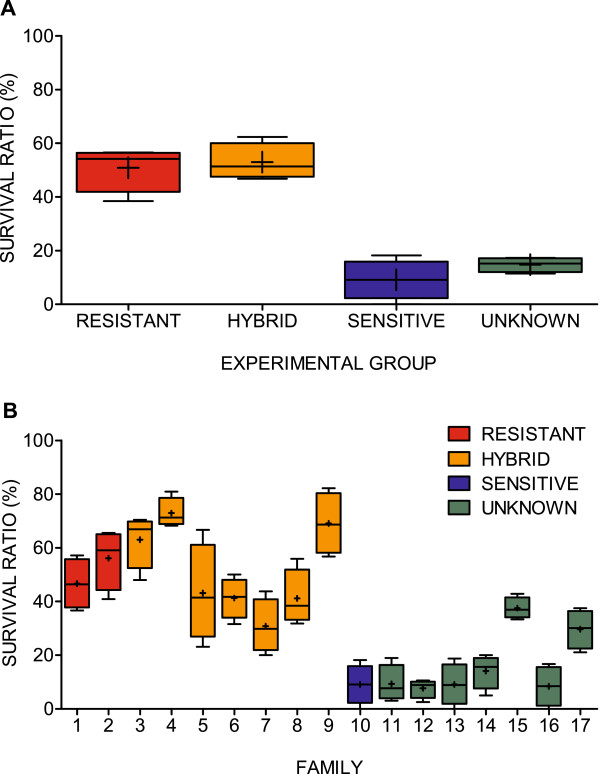
**Variability in survival ratios in the EB trial.** EB challenge results presented as ratios (%) between number of surviving individuals and the total numbers of trial lice separated by the four experimental groups **(A)**, and the 17 families **(B)**. Data presented from the four replicate tanks, where coloured boxes represents the 25-75% quartile, whiskers at min - max, line at median and (+) at mean values.

In total, 37% of the salmon lice (n = 713) survived the EB trial. The survival frequency varied significantly between tanks (χ^2^ = 17.9, 3 df, *P* < 0.001), ranging from 31 to 43% (Table 5). However, adjusting the data for differences in EB exposure time, as described in the Methods section, narrowed the survival range to 34 to 40% (Table 5), and removed this apparent tank effect (χ^2^ = 5.36, 3 df, *P* = 0.15). When survival ratios were viewed at the group level, the resistant and hybrid groups demonstrated a distinct advantage over the sensitive and unknown groups (Figure 3A).

**Table 5:** Summary of common-garden and EB trial results from the replicate tanks

**Tank**	**Common-garden infection**			**EB challenge**			
**ID**	**N**_**0 **_**(cops)**	**n S**	**% S**	**N**_**0 **_**(lice)**	**n S**	**% S**	**% S**_**corr**_
Tank 1	NA	503	11.0	499	216	43.3	39.0
Tank 2	NA	443	9.73	437	153	35.0	33.5
Tank 3	NA	518	11.4	507	193	38.1	39.5
Tank 4	NA	502	11.0	492	151	30.7	35.2
Total	4 554	1 966	43.2	1 935	713	36.8	36.9

N_0_ (cops) = initial number of copepodids at infection; the total number of individuals in the infection batch was split between the four tanks. n S = number of survivors at termination. % S = ratio of survivors divided by the initial numbers of individuals. For the common-garden trial, this is given as percentage of the total number of copepodids (N_0_ (cops)), in the EB challenge as percentage for each tank. N_0_ (lice) = initial number of salmon lice in the one-dose EB trial. % S = survival ratio for exposure time corrected data.

**Figure 4:**
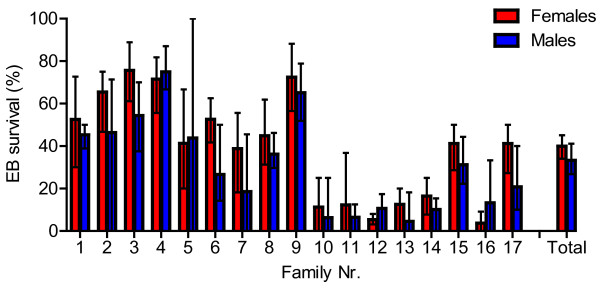
**Gender specific survival ratios in the EB trial.** Survival ratios (%) for females and males, presented by family 1 to 17 and for all individuals, as ‘Total’. Results are averaged over the four replicate tanks, whiskers at min and max values.

Highly significant differences in survival ratios were observed between families (χ^2^ = 432, 16 df, *P* < 0.001). These ranged from 7.9 to 74% in the data pooled across the four replicate tanks (Table 2, Figure 3B). As expected, the survival ratios were relatively high, 46 and 57%, for both families originating from the resistant strain, and low, 8.2%, for the only family with two sensitive parents (Table 2). There was large variability in survival between the hybrid families (Figure 3B), ranging from 30 to 74%. Thus, some of the hybrid families displayed survival higher than the resistant families; while other hybrid families displayed survival lower than the resistant families. When comparing the survival of families from the two hybrid combinations (i.e., resistant mother crossed with sensitive father and vice versa), families of both types were among those displaying higher and lower survival compared to the two families produced from the resistant strain.Variation in survival between the seven families of unknown susceptibility to EB was high (7.9 to 38%). There was, however, a distinct pattern: two families out of seven; Family 15 (38% survivors) and Family 17 (30% survivors) exhibited markedly lower sensitivity to EB (within the lower range for the hybrids’ results) than the other five families, which varied from 7.9 to 9.4% survival, corresponding to the level of the sensitive family, see Figure 3B.Altogether; 40% of the female salmon lice and 33% of the males survived the EB challenge. This was also reflected by higher survival scores for females over males in 14 out of the 17 families (Figure 4).

Model comparison: Judging by the AIC, the best fitted model was obtained when exposure time, family and gender were included as fixed variables (Table 4), the model explained 19.6% of the deviance and a dispersion factor of 1.02 indicated a good model fit. All three variables were highly significant (Anova: *P* < 0.001), but differed in influence; 18.5% of the total deviance was independently explained by family, while 0.5% and 0.4% of the deviance was explained by exposure time and gender, respectively.

As judged by the low difference in AIC (<2) between the models eb.m1 and eb.m2 and between eb.m6 and eb.m7 (Table 4), dividing the hybrid families into two groups by parental combination did not provide a more parsimonious description. Thus, there was no indication whether or not the mechanism involved in EB tolerance is inherited primarily from either the maternal or the paternal side. The logistic regression revealed no relationship (R^2^ = 0.0094, *P* = 0.73) between family clutch size and family survival in the EB trial.

### Change in distribution of families throughout the experiment

**Figure 5:**
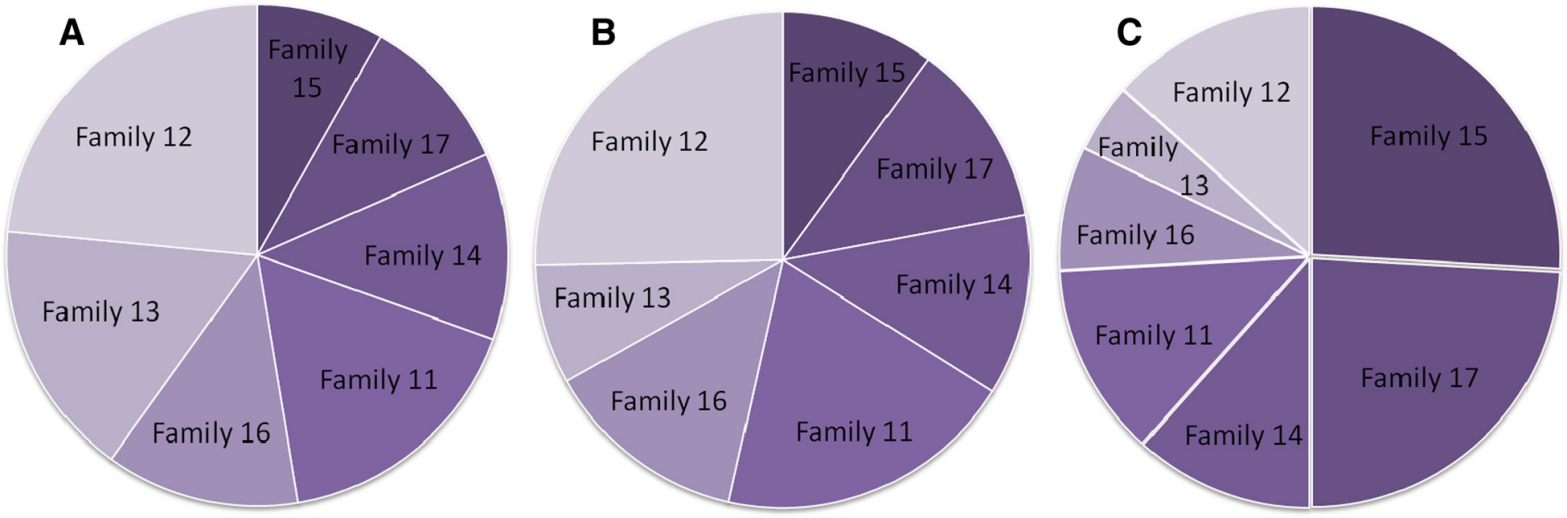
**An illustration of experimental selection, as a proportional shift between families during the experiment.** The change in relative representation of individuals between the seven families defined as ‘unknown’, at different stages of the trials. **A**: Initial number of copepodids from each family at the infection. **B**: Number of lice present at termination of the common-garden trial (34 DPI). **C**: Final proportions of surviving individuals after the EB trial. Families are ranked by their performance in the EB-trial and plotted in falling order, from the darkest slice at 12 o’clock, representing the family scoring highest, and descending clockwise.

Looking at the seven unknown families collected at the farm, at three stages of the experiment; infection (Figure 5A), sampling (Figure 5B) and after termination of the EB trial (Figure 5C), the distribution of family representation in the overall population changed clearly between the start of the experiment and after termination of the EB trial (Figure 5A, C). For example, families 15 and 17 only represented 18% of the 7-family population at the start of the experiment, while at the end of the experiment they represented 50% of the population. Likewise, families 12, 13 and 16 represented 53% of the population at the start of the experiment, while their representation dropped to only 26% upon termination. For both the overall data and the family data, the shifts in distribution of types or families represent the accumulated differential mortality as a result of infection success and survival in the EB trial.

## Discussion

### Development of resistance on fish farms

The development of resistance in target organisms, against farming pesticides or antibiotics in medical practice are examples of human-induced evolutionary changes [79,80]. In food production, resistance to pest control agents represents one of the greatest challenges experienced on a global scale. Cage-based marine salmonid farming is dependent upon chemicals to delouse fish at some stage of the production cycle. This is especially true in regions of high farm density which tend to suffer higher infection levels [81]. Considering the limited number of compounds certified for treatment against sea lice in aquaculture, and the documented emerging resistance of *L. salmonis* to most of these chemicals, this is a critical situation for the continued development of this industry.

Examples of *L. salmonis* displaying resistance, or reduced susceptibility, have been documented for the majority of the chemicals used to delouse fish in commercial fish farms. This includes organophosphates (azamethipos, dichlorvos) [41,82], pyrethroids (cypermethrin, deltamethrin) [44,45], avermectin (emamectin benzoate) [46,48,83] and the disinfectant hydrogen peroxide [43]. Thus far, there have been no records of *L. salmonis* displaying reduced sensitivity or resistance towards the growth inhibitors diflubenzuron and teflubenzuron. However, resistance development against diflubenzuron [84] and target-site mutations conferring resistance towards other chitin inhibitors with similar modes of action [85] have been documented in terrestrial arthropod pests, and it is therefore possible that *L. salmonis* will also develop resistance to diflubenzuron and teflubenzuron.

As a consequence of decreased sensitivity or resistance towards the major chemicals used for delousing on commercial farms, integrated pest management programs for sea lice have been extended to cover the handling and prevention of resistance development to chemicals used at fish farms [86,87]. Key elements of these strategies include: frequent counts of lice on farms, synchronised delousing among farms within regions, rotation of chemical treatments, fallowing areas and the development of bioassays for resistance monitoring. Bioassays, used as pre-treatment tests for assessing functional doses for specific chemicals, and designed to detect increased tolerance towards chemicals before they result in treatment failures at salmon farms, are considered valuable tools in resistance management [88]. Results from bioassays are typically reported as EC_50_ values; which is the concentration of the chemical intended to be used on the farm that will immobilise 50% of the individuals [52,54]. However, while these bioassay techniques are useful for detecting an overall change in tolerance in a group of lice, the underlying genetic structure and the distribution of the sensitivity differences within the group of lice is not necessarily directly inferred from the EC_50_ value itself [89]. Thus, farms are at risk of using chemicals to delouse groups of fish when the bioassay results give a satisfactory sensitivity level on a sample of lice collected from the fish prior to treatment, but due to the underlying distribution of reduced sensitivity in the population, may still be leading to increased resistance. This situation has been illustrated in the present study where clear differences in susceptibility to EB were observed among families of lice originating from the farm located on Strøno, despite the fact that the farm had not reported any treatment failures. Possible consequences of this were demonstrated by the pronounced shift in family representation in the group of lice originating from this farm, before and after the single dose trial was conducted (Figure 5). Extrapolating our results from the laboratory to the farm, these results illustrate how the development of resistance can occur on a commercial farm, even in the apparent absence of treatment failure in the first instance. Repeated treatments on that farm would have likely resulted in a gradual increase in the EC_50_ value where the frequency of lice displaying the resistance allele(s) would have increased. While the experimental system presented here does not intend to replace bioassays used for routine testing within the aquaculture industry, it nevertheless enables the opportunity to investigate the underlying distribution of heritable variability for chemical resistance within lice.

### Fitness implications of EB resistance

The present study was not designed to specifically address potential fitness consequences associated with decreased sensitivity to EB. However, there was no evidence to suggest lower infection success or reduced clutch sizes in families displaying a higher degree of EB tolerance than in more sensitive families. Therefore, for these specific traits, and under the described experimental conditions, no fitness cost associated with EB tolerance was observed in this study. This is consistent with results from an experiment where no significant differences in reproductive output, measured as hatching success and copepodid survival, were detected between a sensitive, a resistant, and a hybrid strain of *L. salmonis* reared for multiple generations [48]. Furthermore, in that study, no decrease of EB tolerance was detected over multiple generations in the absence of EB-based selection. Thus, taken together, none of the results from these two studies suggest that development of EB resistance in *L. salmonis* is associated with a clear cost in fitness. For the other chemical groups where decreased sensitivity or resistance has been documented in *L. salmonis*, there is little knowledge at present regarding potential fitness costs associated with resistance. However, the azamethipos-resistant type of the enzyme acetylcholinesterase has been confirmed in *L. salmonis* 3*–*5 years after the use of organophosphates was discontinued in the region [82], which suggests a relatively low fitness costs for this specific resistance mechanism.

Resistance development and potential fitness costs of resistance to pesticides have been extensively studied in insects. There are examples where resistance development is associated with clear fitness costs [90,91] as well as studies where no fitness costs could be detected [92,93]. Turning back to the salmonid farming industry and EB resistance in *L. salmonis*, the practical consequences of a ‘low fitness cost’ scenario could be that even in the absence of direct selection pressure, emerging genotypes for decreased sensitivity or resistance to EB may prevail in sea lice populations for many generations after the last treatment. This might render the chemical useless for a long period of time. Also, this could serve as the backdrop for the emergence of multiresistance if, as part of a treatment rotation practice, another delousing substance is taken into use while part of the regional lice population still carry traits of EB tolerance. Furthermore, given the dispersal potential of *L. salmonis*, manifested by the species’ weak population genetic structuring throughout the Atlantic [70,94,95], it is likely that the mutations causing the resistance may quickly spread between regions, further complicating the management of resistance development. Thus, the aquaculture industry needs to ensure correct application of this chemical to delouse commercial salmon farms in order to prolong its usable life, which should include more detailed surveys of tolerance levels than bioassays alone.

### Experimental system functionality and implications for studying evolutionary processes in lice

Evolutionary questions in parasites are notoriously difficult to address, in part due to the lack of suitable experimental systems in which it is possible to conduct reproducible experiments. Firstly, such systems require the ability to control the life-cycle of the parasite under experimental conditions. This involves both parasite and host-specific considerations. Secondly, such a system must have the potential to control and manipulate the parasites’ environment, and at the same time control reproduction and mating in order to be able to evaluate fitness and adaptation under different conditions. The experimental system presented here fulfils these critical elements, and thus, represents a significant advance to address evolutionary questions in *L. salmonis*. Furthermore, it highlights the opportunity to build similar systems to address evolutionary questions in similar copepods.

The number of *L. salmonis* families produced and used in the current experiment was constrained by numbers of experimental tanks, the numbers of hosts that could be accommodated in each tank, and not least, animal welfare considerations regarding the maximum number of *L. salmonis* permitted per host. Thus, by choosing to include five experimental groups of *L. salmonis* in the experimental set-up, the number of families was low for some of these groups. Specifically the ‘sensitive’ strain consisted only of one family that also displayed a low infection success, and the resistant strain was only represented by two families. As a result, this challenged quantitative comparisons between some of the strains. Nevertheless, the main focus of this study was to construct and test an experimental system for comparing differences between families, which was demonstrated with high accuracy. This was the first attempt to combine these methods, and the noted challenges could be avoided in subsequent studies by using larger tanks with more host fish in them, or alternatively, by restricting the number of strains to be investigated in each trial.

Despite the somewhat unbalanced distribution of families between experimental groups, the system demonstrated its potential to uncover relationships between phenotype and genotype by displaying differences in infection success and EB tolerance between families of *L. salmonis*. There is a range of other evolutionary questions linked with this and similar parasites which are now accessible via this or similar experimental setups. For copepods in general, very little is known about their capacity to evolve in response to changing environmental or ecological conditions [96]. While farming-induced evolutionary changes in life-history and virulence in *L. salmonis* have been suggested [61], and adaptations to other environmental parameters such as temperature and salinity may exist in this and similar species, these remain to be addressed and validated. Furthermore, genetic differences in susceptibility to *L. salmonis* have been detected between different families [36,97], populations [98,99] and species of salmonids [100,101]. While much of these differences are likely to reflect differences between host individuals in their ability to reject the parasite [101,102], the experimental system described here also permits the ability to develop experiments evaluating for example host preference of lice families produced from different strains adapted to different host types.

## Conclusions

Overall, the different components of the experimental setup performed in a robust manner, which permitted for the first time, quantification of phenotypic differences in infection and chemical tolerance parameters among strains and families of *L. salmonis*. To our knowledge, this represents the first study where families of any multi-cellular parasite have been established and compared in performance under communal rearing conditions in a so-called common-garden experiment design. This was achieved by combining predictable and well-established full life-cycle rearing systems [63], production of families where both paternal and maternal contribution was controlled from strains with defined backgrounds in EB tolerance, and high throughput microsatellite genotyping [94] in order to conduct parentage testing. This represents a major advance in the tools available to study evolutionary questions in *L. salmonis*, and other caligid copepods in general.

## Availability of supporting data

The data set supporting the results of this article is available in the Dryad Digital Repository: http://dx.doi.org/10.5061/dryad.923n7[103] and included within the article’s additional files (see Additional file 3: Table S2).

## Abbreviations

EB: Emamectin benzoate; DNA: Deoxyribonucleic acid; IMR: Institute of marine research; DPI: Days post infection; DDPI: Degree days post infection; PCR: Polymerase chain reaction; AIC: Akaike’s information criterion.

## Competing interests

The authors declare that they have no competing interests.

## Authors’ contributions

LL maintained and conducted the majority of the experimental part of the study, carried out the molecular and statistical analyses, participated in planning the study and drafted the first version of the manuscript together with KG. KG also had the main responsibility for study design and coordination, participated in the practical conduction of the experiments and data interpretation. PGE carried out the one-dose EB challenge, participated in the conduction of experiments, and contributed to the manuscript. FN participated in the design of the study, data interpretation and contributed to the manuscript. MSM supervised and participated in the statistical analysis, contributed to interpretation of data and to the manuscript. All authors read and approved the final manuscript.

## Supplementary Material

Additional file 1 **Tank effects in EB survival data redundant of exposure time.** In the EB trial, the exposure times differed for the four tank replicates. In order to assess whether there was a tank effect independent of exposure time in the trial results, a logistic regression mixed model was formulated, with exposure time, gender of individuals and experimental group as fixed variables and family as a random factor. Here, the Pearson residuals of this model are plotted against tanks. Click here for file

Additional file 2 **Ranked GLMs and GLMMs for infection success data when families 10 and 13 were excluded.** AIC-based model comparison for the infection success results revealed a significant, albeit low (>2%) family effect in the common-garden trial (Table 4). Presented here is a comparison of the same models when the two families with distinctly lower infection success (Family 10 and Family 13) were excluded from the dataset. Click here for file

Additional file 3 **Background data and results.** Background data and results for production of families in single tanks and for all individual lice sampled from the common-garden infection. Click here for file

## References

[B1] FAO Fisheries and Aquaculture Department: Fishery Statistical Collections[http://www.fao.org/fishery/statistics/global-aquaculture-production/en]

[B2] Fiskeridirektoratet Nøkkeltall fra norsk havbruksnæring 2012. Report from The Norwegian Directorate of Fisheries In Norwegian. [http://www.fiskeridir.no/content/download/30475/266778/version/3/file/nokkeltall-havbruk-2012.pdf]

[B3] Glover KA Quintela M Wennevik V Besnier F Sørvik AGE Skaala Ø Three decades of farmed escapees in the wild: A spatio-temporal analysis of Atlantic salmon population genetic structure throughout Norway PLoS One 2012 7 8 e43129 10.1371/journal.pone.0043129 22916215 PMC3419752

[B4] Glover K Pertoldi C Besnier F Wennevik V Kent M Skaala Ø Atlantic salmon populations invaded by farmed escapees: quantifying genetic introgression with a Bayesian approach and SNPs Bmc Genetics 2013 14 1 74 10.1186/1471-2156-14-74 23968202 PMC3765417

[B5] Skaala Ø Wennevik V Glover KA Evidence of temporal genetic change in wild Atlantic salmon, *Salmo salar* L., populations affected by farm escapees ICES J Mar Sci 2006 63 7 1224 1233 10.1016/j.icesjms.2006.04.005

[B6] Håstein T Lindstad T Diseases in wild and cultured salmon – possible interaction Aquaculture 1991 98 1–3 277 288

[B7] Costello MJ The global economic cost of sea lice to the salmonid farming industry J Fish Dis 2009 32 1 115 118 10.1111/j.1365-2761.2008.01011.x 19245636

[B8] Johnson SC Treasurer JW Bravo S Nagasawa K Kabata Z A review of the impact of parasitic copepods on marine aquaculture Zool Stud 2004 43 2 229 243

[B9] Costello MJ How sea lice from salmon farms may cause wild salmonid declines in Europe and North America and be a threat to fishes elsewhere Proc R Soc B-Biol Sci 2009 276 1672 3385 3394 10.1098/rspb.2009.0771 PMC2817184 19586950

[B10] Pike AW Wadsworth SL **Sealice on salmonids: Their biology and control** AdvParasitol 1999 44 233 337 10.1016/s0065-308x(08)60233-x 10563397

[B11] Bjørn PA Finstad B Kristoffersen R Salmon lice infection of wild sea trout and Arctic char in marine and freshwaters: the effects of salmon farms Aquaculture Res 2001 32 12 947 962 10.1046/j.1365-2109.2001.00627.x

[B12] Bjørn PA Finstad B Salmon lice, *Lepeophtheirus salmonis* (Krøyer), infestation in sympatric populations of Arctic char, *Salvelinus alpinus* (L.), and sea trout, *Salmo trutta* (L.), in areas near and distant from salmon farms ICES J Mar Sci 2002 59 1 131 139 10.1006/jmsc.2001.1143

[B13] Tully O Gargan P Poole WR Whelan KF Spatial and temporal variation in the infestation of sea trout (*Salmo trutta* L.) by the caligid copepod *Lepeophtheirus salmonis* (Krøyer) in relation to sources of infection in Ireland Parasitology 1999 119 41 51 10.1017/S003118209900445X 10446703

[B14] Krkošek M Lewis MA Volpe JP Transmission dynamics of parasitic sea lice from farm to wild salmon Proc R Soc B-Biol Sci 2005 272 1564 689 696 10.1098/rspb.2004.3027 PMC1602048 15870031

[B15] Price MHH Morton A Reynolds JD Evidence of farm-induced parasite infestations on wild juvenile salmon in multiple regions of coastal British Columbia, Canada Can J Fish Aquat Sci 2010 67 12 1925 1932 10.1139/F10-105

[B16] Tully O Whelan KF Production of nauplii of Lepeophtheirus salmonis (Krøyer) (Copepoda, Caligidae) from farmed and wild salmon and its relation to the infestation of wild sea trout (Salmo trutta L) off the west coast of Ireland in 1991 Fish Res 1993 17 1–2 187 200

[B17] Butler JRA Watt J Assessing and managing the impacts of marine salmon farms on wild Atlantic salmon in western Scotland: Identifying priority rivers for conservation Salmon at the Edge 2007 Oxford, UK: Blackwell Science Ltd 93 118

[B18] Gargan PG Tully O Poole WR Relationship between sea lice infestation, sea lice production and sea trout survival in Ireland, 1992–2001 Salmon at the Edge 2007 Oxford, UK: Blackwell Science Ltd 119 135

[B19] Krkošek M Ford JS Morton A Lele S Myers RA Lewis MA Declining wild salmon populations in relation to parasites from farm salmon Science 2007 318 5857 1772 1775 10.1126/science.1148744 18079401

[B20] Ford JS Myers RA A global assessment of salmon aquaculture impacts on wild salmonids PLOS Biol 2008 6 2 e33 10.1371/journal.pbio.0060033 18271629 PMC2235905

[B21] Kabata Z Parasitic copepoda of British Fishes 1979 London: The Ray Society

[B22] Kabata Z Copepods parasitic on fishes: keys and notes for the identification of British species 2003 Shrewsbury: Published for the Linnean Society of London and the Estuarine and Brackish-Water Sciences Association by Field Studies Council

[B23] Skern-Mauritzen R Torrissen O Glover K Pacific and Atlantic *Lepeophtheirus salmonis* (Krøyer, 1838) are allopatric subspecies: *Lepeophtheirus salmonis salmonis* and *L. salmonis oncorhynchi* subspecies novo BMC Genet 2014 15 1 32 10.1186/1471-2156-15-32 24628716 PMC4007600

[B24] Yazawa R Yasuike M Leong J von Schalburg KR Cooper GA Beetz-Sargent M Robb A Davidson WS Jones SRM Koop BF EST and mitochondrial DNA sequences support a distinct Pacific form of salmon louse, Lepeophtheirus salmonis Mar Biotechnol 2008 10 6 741 749 10.1007/s10126-008-9112-y 18574633

[B25] Mordue AJ Birkett MA A review of host finding behaviour in the parasitic sea louse, Lepeophtheirus salmonis (Caligidae: Copepoda) J Fish Dis 2009 32 1 3 13 10.1111/j.1365-2761.2008.01004.x 19245627

[B26] Johnson SC Albright LJ The developmental stages of Lepeophtheirus salmonis (Krøyer, 1837) (Copepoda, Caligidae) Can J Zool 1991 69 4 929 950 10.1139/z91-138

[B27] Hamre LA Eichner C Caipang CMA Dalvin ST Bron JE Nilsen F Boxshall G Skern-Mauritzen R The Salmon louse Lepeophtheirus salmonis (Copepoda: Caligidae) life cycle has only two chalimus stages PLoS One 2013 8 9 e73539 10.1371/journal.pone.0073539 24069203 PMC3772071

[B28] Heuch PA Nordhagen JR Schram TA Egg production in the salmon louse Lepeophtheirus salmonis (Krøyer) in relation to origin and water temperature Aquaculture Res 2000 31 11 805 814 10.1046/j.1365-2109.2000.00512.x

[B29] Costello MJ Boxshall GA, Defaye D Review of methods to control sea lice (Caligidae: Crustacea) infestation on salmon (*Salmo salar*) farms Pathogens of wild and farmed fish: sea lice 1993 Chichester, UK: Ellis Horwood 219 252

[B30] Ritchie G Boxaspen KK Jones S, Beamish R Salmon louse management on farmed salmon—Norway Salmon Lice: An Integrated Approach to Understanding Parasite Abundance and Distribution 2011 Wiley-Blackwell 151 176

[B31] Werkman M Green DM Murray AG Turnbull JF The effectiveness of fallowing strategies in disease control in salmon aquaculture assessed with an SIS model Prev Vet Med 2011 98 1 64 73 10.1016/j.prevetmed.2010.10.004 21040988

[B32] Bjørn PA Sivertsgård R Finstad B Nilsen R Serra-Llinares RM Kristoffersen R Area protection may reduce salmon louse infection risk to wild salmonids Aquaculture Environ Interact 2011 1 3 233 244 10.3354/aei00023

[B33] Serra-Llinares RM Bjørn PA Finstad B Nilsen R Harbitz A Berg M Asplin L Salmon lice infection on wild salmonids in marine protected areas: an evaluation of the Norwegian ‘National Salmon Fjords’ Aquaculture Enviro Interact 2014 5 1 1 16 10.3354/aei00090

[B34] Treasurer JW A review of potential pathogens of sea lice and the application of cleaner fish in biological control Pest Manag Sci 2002 58 6 546 558 10.1002/ps.509 12138621

[B35] Skiftesvik AB Bjelland RM Durif CMF Johansen IS Browman HI Delousing of Atlantic salmon (*Salmo salar*) by cultured vs. wild ballan wrasse (*Labrus bergylta*) Aquaculture 2013 402 113

[B36] Glover KA Aasmundstad T Nilsen F Storset A Skaala Ø Variation of Atlantic salmon families (*Salmo salar* L.) in susceptibility to the sea lice *Lepeophtheirus salmonis* and *Caligus elongatus* Aquaculture 2005 245 1–4 19 30

[B37] Gjerde B Odegard J Thorland I Estimates of genetic variation in the susceptibility of Atlantic salmon (Salmo salar) to the salmon louse Lepeophtheirus salmonis Aquaculture 2011 314 1–4 66 72

[B38] Raynard RS Bricknell IR Billingsley PF Nisbet AJ Vigneau A Sommerville C Development of vaccines against sea lice Pest Manag Sci 2002 58 6 569 575 10.1002/ps.474 12138623

[B39] Frost P Nilsen F Validation of reference genes for transcription profiling in the salmon louse, Lepeophtheirus salmonis, by quantitative real-time PCR Vet Parasitol 2003 118 1–2 169 174 14651887 10.1016/j.vetpar.2003.09.020

[B40] Burridge L Weis JS Cabello F Pizarro J Bostick K Chemical use in salmon aquaculture: A review of current practices and possible environmental effects Aquaculture 2010 306 1–4 7 23

[B41] Jones MW Sommerville C Wootten R Reduced sensitivity of the salmon louse, Lepeophtheirus salmonis, to the organophosphate dichlorvos J Fish Dis 1992 15 2 197 202 10.1111/j.1365-2761.1992.tb00654.x

[B42] Roth M Richards RH Dobson DP Rae GH Field trials on the efficacy of the organophosphorus compound azamethiphos for the control of sea lice (Copepoda: Caligidae) infestations of farmed Atlantic salmon (Salmo salar) Aquaculture 1996 140 3 217 239 10.1016/0044-8486(95)01181-1

[B43] Treasurer JW Wadsworth S Grant A Resistance of sea lice, Lepeophtheirus salmonis (Krøyer), to hydrogen peroxide on farmed Atlantic salmon, Salmo salar L Aquaculture Res 2000 31 11 855 860 10.1046/j.1365-2109.2000.00517.x

[B44] Fallang A Denholm I Horsberg TE Williamson MS Novel point mutation in the sodium channel gene of pyrethroid-resistant sea lice Lepeophtheirus salmonis (Crustacea : Copepoda) Dis Aquat Organ 2005 65 2 129 136 16060266 10.3354/dao065129

[B45] Sevatdal S Horsberg TE Kartlegging av pyretroidresistens hos lakselus Norsk Fiskeoppdrett 2000 12 34 35

[B46] Lees F Baillie M Gettinby G Revie CW The efficacy of emamectin benzoate against Infestations of *Lepeophtheirus salmonis* on farmed Atlantic salmon (*Salmo salar* L) in Scotland, 2002–2006 PLoS One 2008 3 2 e1549 10.1371/journal.pone.0001549 18253496 PMC2212131

[B47] Igboeli OO Fast MD Heumann J Burka JF Role of P-glycoprotein in emamectin benzoate (SLICE®) resistance in sea lice, Lepeophtheirus salmonis Aquaculture 2012 344 40 47

[B48] Espedal PG Glover KA Horsberg TE Nilsen F Emamectin benzoate resistance and fitness in laboratory reared salmon lice (Lepeophtheirus salmonis) Aquaculture 2013 416–417 111 118

[B49] Horsberg TE Avermectin Use in Aquaculture Curr Pharmaceut Biotechnol 2012 13 6 1095 1102 10.2174/138920112800399158 22039799

[B50] Mattilsynet **Lakselus statusrapport 9** Report from the Norwegian Food Authority 2010 5 2 7 In Norwegian

[B51] Hoy MA Myths, models and mitigation of resistance to pesticides Phil Trans Roy Soc Lond B Biol Sci 1998 353 1376 1787 1795 10.1098/rstb.1998.0331 10021775 PMC1692395

[B52] Sevatdal S Horsberg TE Determination of reduced sensitivity in sea lice (Lepeophtheirus salmonis Krøyer) against the pyrethroid deltamethrin using bioassays and probit modelling Aquaculture 2003 218 1–4 21 31

[B53] Westcott JD Stryhn H Burka JF Hammell KL Optimization and field use of a bioassay to monitor sea lice Lepeophtheirus salmonis sensitivity to emamectin benzoate Dis Aquat Organ 2008 79 2 119 131 18500028 10.3354/dao01887

[B54] SEARCH Consortium Sea lice resistance to chemotherapeutants: A handbook in resistance management 2006 2 52 EU Research Project

[B55] Houde ALS Fraser DJ O’Reilly P Hutchings JA Maternal and paternal effects on fitness correlates in outbred and inbred Atlantic salmon (Salmo salar) Can J Fish Aquat Sci 2011 68 3 534 549 10.1139/f2011-001

[B56] Fraser DJ Cook AM Eddington JD Bentzen P Hutchings JA Mixed evidence for reduced local adaptation in wild salmon resulting from interbreeding with escaped farmed salmon: complexities in hybrid fitness Evol Appl 2008 1 3 501 512 10.1111/j.1752-4571.2008.00037.x PMC3352379 25567731

[B57] Solberg MF Zhang Z Nilsen F Glover KA Growth reaction norms of domesticated, wild and hybrid Atlantic salmon families in response to differing social and physical environments BMC Evol Biol 2013 13 234 10.1186/1471-2148-13-234 24165438 PMC4231500

[B58] Skaala Ø Glover KA Barlaup BT Svasand T Besnier F Hansen MM Borgstrom R Performance of farmed, hybrid, and wild Atlantic salmon (Salmo salar) families in a natural river environment Can J Fish Aquat Sci 2012 69 12 1994 2006 10.1139/f2012-118

[B59] McGinnity P Prodohl P Maoileidigh NO Hynes R Cotter D Baker N O’Hea B Ferguson A Differential lifetime success and performance of native and non-native Atlantic salmon examined under communal natural conditions J Fish Biol 2004 65 173 187 10.1111/j.0022-1112.2004.00557.x

[B60] McGinnity P Stone C Taggart JB Cooke D Cotter D Hynes R McCamley C Cross T Ferguson A Genetic impact of escaped farmed Atlantic salmon (*Salmo salar* L.) on native populations: use of DNA profiling to assess freshwater performance of wild, farmed, and hybrid progeny in a natural river environment ICES J Mar Sci 1997 54 6 998 1008

[B61] Mennerat A Hamre L Ebert D Nilsen F Davidova M Skorping A Life history and virulence are linked in the ectoparasitic salmon louse Lepeophtheirus salmonis J Evol Biol 2012 25 5 856 861 10.1111/j.1420-9101.2012.02474.x 22356541

[B62] Hamre LA Nilsen F Individual fish tank arrays in studies of Lepeophtheirus salmonis and lice loss variability Dis Aquat Organ 2011 97 1 47 56 10.3354/dao02397 22235594

[B63] Hamre LA Glover KA Nilsen F Establishment and characterisation of salmon louse (Lepeophtheirus salmonis (Krøyer 1837)) laboratory strains Parasitol Int 2009 58 4 451 460 10.1016/j.parint.2009.08.009 19732850

[B64] WHO Expert Committee on Malaria, Seventh Report WHO Technical Report Series No 125 1958 Geneva Switzerland: World Health Organization 13636243

[B65] Whalon ME Mota-Sanchez D Hollingworth RM Whalon ME, Mota-Sanchez D, Hollingworth RM **Analysis of global pesticide resistance in arthropods** Global pesticide resistance in arthropods 2008 Oxfordshire: CABI International 5 31

[B66] Brent KJ National Research Council, Committee on Strategies for the Management of Pesticide Resistant Pest Populations Detection and monitoring of resistant forms: An overview Pesticide Resistance: Strategies and Tactics for Management 1986 Washington, D.C: National Academy Press

[B67] Todd CD Stevenson RJ Reinardy H Ritchie MG Polyandry in the ectoparasitic copepod Lepeophtheirus salmonis despite complex precopulatory and postcopulatory mate-guarding Mar Ecol-Prog Ser 2005 303 225 234

[B68] Ritchie G Mordue AJ Pike AW Rae GH Observations on mating and reproductive behaviour of Lepeophtheirus salmonis, Krøyer (Copepoda: Caligidae) J Exp Mar Biol Ecol 1996 201 1–2 285 298

[B69] Taggart JB FAP: an exclusion-based parental assignment program with enhanced predictive functions Mol Ecol Notes 2007 7 3 412 415

[B70] Todd CD Walker AM Ritchie MG Graves JA Walker AF Population genetic differentiation of sea lice (Lepeophtheirus salmonis) parasitic on Atlantic and Pacific salmonids: analyses of microsatellite DNA variation among wild and farmed hosts Can J Fish Aquat Sci 2004 61 7 1176 1190 10.1139/f04-069

[B71] Messmer AM Rondeau EB Jantzen SG Lubieniecki KP Davidson WS Koop BF Assessment of population structure in Pacific Lepeophtheirus salmonis (Krøyer) using single nucleotide polymorphism and microsatellite genetic markers Aquaculture 2011 320 3–4 183 192

[B72] Nolan DV Martin SAM Kelly Y Glennon K Palmer R Smith T McCormack GP Powell R Development of microsatellite PCR typing methodology for the sea louse, Lepeophtheirus salmonis (Krøyer) Aquaculture Res 2000 31 11 815 822 10.1046/j.1365-2109.2000.00514.x

[B73] Nolan DV Powell R Geographic and temporal genetic structure in Lepeophtheirus salmonis from four salmon farms along the northwest and west coasts of Ireland: results from a microsatellite analysis Hydrobiologia 2009 617 55 63 10.1007/s10750-008-9525-7

[B74] Zuur AF Ieno EN Walker NJ Saveliev AA Smith GM Mixed effects models and extensions in ecology with R 2009 New York, NY: Springer

[B75] Burnham KP Anderson DR Model selection and multimodel inference: A practical information-theoretic approach 2002 New York, NY: Springer-Verlag

[B76] Zuur AF Saveliev AA Ieno EN Zero inflated models and Generalized Linear Mixed Models with R 2012 Highland Statistics Ltd

[B77] R A Language and Environment for Statistical Computing [http://www.R-project.org/]

[B78] lme4 Linear mixed-effects models using Eigen and S4. R package version 1.0-5 [http://lme4.r-forge.r-project.org/]

[B79] Lebarbenchon C Brown SP Poulin R Gauthier-Clerc M Thomas F Evolution of pathogens in a man-made world Mol Ecol 2008 17 1 475 484 10.1111/j.1365-294X.2007.03375.x 18173509 PMC7168490

[B80] Palumbi SR Evolution - Humans as the world’s greatest evolutionary force Science 2001 293 5536 1786 1790 10.1126/science.293.5536.1786 11546863

[B81] Jansen PA Kristoffersen AB Viljugrein H Jimenez D Aldrin M Stien A Sea lice as a density-dependent constraint to salmonid farming Proc R Soc B-Biol Sci 2012 279 1737 2330 2338 10.1098/rspb.2012.0084 PMC3350688 22319130

[B82] Fallang A Ramsay JM Sevatdal S Burka JF Jewess P Hammell KL Horsberg TE Evidence for occurrence of an organophosphate-resistant type of acetylcholinesterase in strains of sea lice (Lepeophtheirus salmonis Krøyer) Pest Manag Sci 2004 60 12 1163 1170 10.1002/ps.932 15578596

[B83] Jones PG Hammell KL Dohoo IR Revie CW Effectiveness of emamectin benzoate for treatment of Lepeophtheirus salmonis on farmed Atlantic salmon Salmo salar in the Bay of Fundy, Canada Dis Aquat Organ 2012 102 1 53 64 10.3354/dao02517 23209078

[B84] Oppenoorth FJ Vanderpas LJT Cross-resistance to diflubenzuron in resistant strains of housefly, *Musca domestica* Entomol Exp Appl 1977 21 3 217 228 10.1111/j.1570-7458.1977.tb02675.x

[B85] Van Leeuwen T Demaeght P Osborne EJ Dermauw W Gohlke S Nauen R Grbić M Tirry L Merzendorfer H Clark RM Population bulk segregant mapping uncovers resistance mutations and the mode of action of a chitin synthesis inhibitor in arthropods Proc Natl Acad Sci 2012 109 12 4407 4412 10.1073/pnas.1200068109 22393009 PMC3311382

[B86] FKD Forskrift om bekjempelse av lus i akvakulturanlegg Regulation text from the Norwegian Ministry of Fisheries and Coastal Affairs 2012 13 In Norwegian. [http://lovdata.no/dokument/SF/forskrift/2012-12-05-1140]

[B87] New Brunswick Integrated Pest Management Plan for Sea Lice [http://www.dfo-mpo.gc.ca/aquaculture/consultations/2012/RASRR-NB-eng.htm]

[B88] Denholm I Devine GJ Horsberg TE Sevatdal S Fallang A Nolan DV Powell R Analysis and management of resistance to chemotherapeutants in salmon lice, Lepeophtheirus salmonis (Copepoda: Caligidae) Pest Manag Sci 2002 58 6 528 536 10.1002/ps.482 12138619

[B89] Denholm I Monitoring and interpreting changes in insecticide resistance Funct Ecol 1990 4 5 601 608 10.2307/2389727

[B90] Berticat C Bonnet J Duchon S Agnew P Weill M Corbel V Costs and benefits of multiple resistance to insecticides for *Culex quinquefasciatus* mosquitoes BMC Evol Biol 2008 8 104 10.1186/1471-2148-8-104 18397515 PMC2359736

[B91] Bourguet D Guillemaud T Chevillon C Raymond M Fitness costs of insecticide resistance in natural breeding sites of the mosquito Culex pipiens Evolution 2004 58 1 128 135 10.1111/j.0014-3820.2004.tb01579.x 15058725

[B92] Bielza P Quinto V Gravalos C Abellan J Fernandez E Lack of fitness costs of insecticide resistance in the western flower thrips (Thysanoptera: Thripidae) J Econ Entomol 2008 101 2 499 503 10.1603/0022-0493(2008)101[499:LOFCOI]2.0.CO;2 18459417

[B93] McCart C Buckling A, ffrench-Constant RH: DDT resistance in flies carries no cost Curr Biol 2005 15 15 R587 R589 10.1016/j.cub.2005.07.054 16085476

[B94] Glover KA Stølen ÅB Messmer A Koop BF Torrissen O Nilsen F Population genetic structure of the parasitic copepod Lepeophtheirus salmonis throughout the Atlantic Mar Ecol-Prog Ser 2011 427 161 172

[B95] Tjensvoll K Glover KA Nylund A Sequence variation in four mitochondrial genes of the salmon louse Lepeophtheirus salmonis Dis Aquat Organ 2006 68 3 251 259 16610591 10.3354/dao068251

[B96] Bron JE Frisch D Goetze E Johnson SC Lee CE Wyngaard GA Observing copepods through a genomic lens Front Zool 2011 8 22 10.1186/1742-9994-8-22 21933388 PMC3184258

[B97] Kolstad K Heuch PA Gjerde B Gjedrem T Salte R Genetic variation in resistance of Atlantic salmon (Salmo salar) to the salmon louse Lepeophtheirus salmonis Aquaculture 2005 247 1–4 145 151

[B98] Glover KA Nilsen F Skaala Ø Taggart JB Teale AJ Differences in susceptibility to sea lice infection between a sea run and a freshwater resident population of brown trout J Fish Biol 2001 59 6 1512 1519 10.1111/j.1095-8649.2001.tb00216.x

[B99] Glover KA Skaala O Nilsen F Olsen R Teale AJ Taggart JB Differing susceptibility of anadromous brown trout (*Salmo trutta* L.) populations to salmon louse (*Lepeophtheirus salmonis* (Krøyer, 1837)) infection ICES J Mar Sci 2003 60 5 1139 1148 10.1016/S1054-3139(03)00088-2

[B100] Fast MD Ross NW Mustafa A Sims DE Johnson SC Conboy GA Speare DJ Johnson G Burka JF Susceptibility of rainbow trout Oncorhynchus mykiss, Atlantic salmon Salmo salar and coho salmon Oncorhynchus kisutch to experimental infection with sea lice Lepeophtheirus salmonis Dis Aquat Organ 2002 52 1 57 68 12517006 10.3354/dao052057

[B101] Johnson SC Albright LJ Comparative susceptibility and histopathology of the response of naive Atlantic, Chinook and Coho salmon to experimental infection with Lepeophtheirus salmonis (Copepoda, Caligidae) Dis Aquat Organ 1992 14 3 179 193

[B102] Johnson SC Albright LJ Effects of cortisol implants on the susceptibility and the histopathology of the responses of naive coho salmon Oncorhynchus kisutch to experimental infection with Lepeophtheirus salmonis (Copepoda, Caligidae) Dis Aquat Organ 1992 14 3 195 205

[B103] Ljungfeldt LER Espedal PG Nilsen F Skern-Mauritzen M Glover KA Common-garden and EB trial data from 17 families of Lepeophtheirus salmonis Dryad Digital Repository 2014 http://dx.doi.org/10.5061/dryad.923n7

