# Supplementary figures and images for: A common-garden experiment to quantify evolutionary processes in copepods: the case of emamectin benzoate resistance in the parasitic sea louse Lepeophtheirus salmonis
Source: BMC Evol Biol. 2014 May 19;14:108. doi: 10.1186/1471-2148-14-108 (PMC4057923; doi:10.1186/1471-2148-14-108)

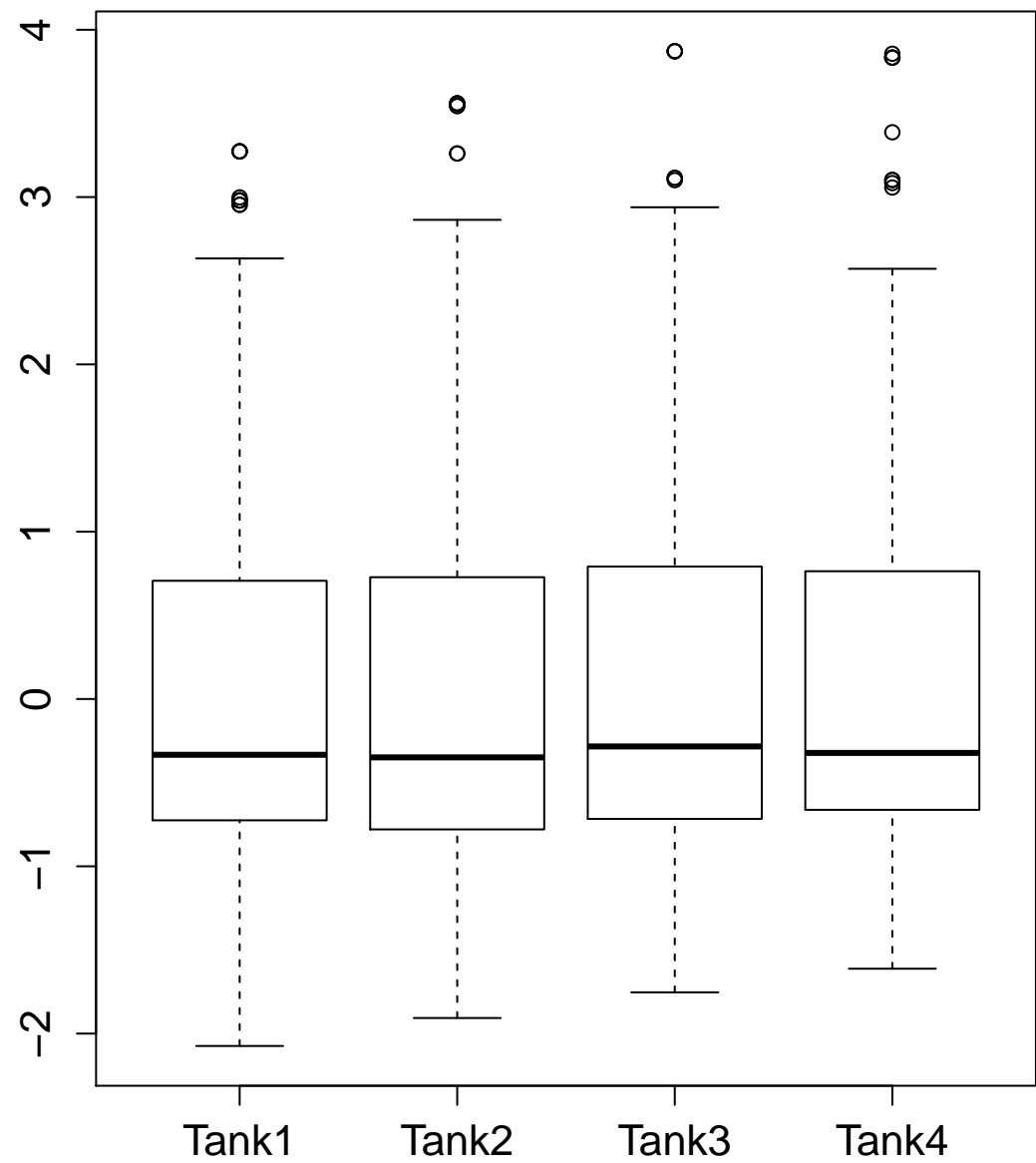

Supplement: Additional file 1 — Tank effects in EB survival data redundant of exposure time. In the EB trial, the exposure times differed for the four tank replicates. In order to assess whether there was a tank effect independent of exposure time in the trial results, a logistic regression mixed model was formulated, with exposure time, gender of individuals and experimental group as fixed variables and family as a random factor. Here, the Pearson residuals of this model are plotted against tanks. [file 1471-2148-14-108-S1.pdf]
