# Supplementary material for: A common-garden experiment to quantify evolutionary processes in copepods: the case of emamectin benzoate resistance in the parasitic sea louse Lepeophtheirus salmonis
Source: BMC Evol Biol. 2014 May 19;14:108. doi: 10.1186/1471-2148-14-108 (PMC4057923; doi:10.1186/1471-2148-14-108)
Supplement: Additional file 2 — Ranked GLMs and GLMMs for infection success data when families 10 and 13 were excluded. AIC-based model comparison for the infection success results revealed a significant, albeit low (>2%) family effect in the common-garden trial (Table 4). Presented here is a comparison of the same models when the two families with distinctly lower infection success (Family 10 and Family 13) were excluded from the dataset. [file 1471-2148-14-108-S2.pdf]

Additional file 2. Ranked GLMs and GLMMs for infection success data if Family 10 and Family 13 were excluded from the analysis.

Model	Fixed effect	Random effect	df	AIC	ΔAIC
Cops15.m3	Dam	Family	4	5 606.2	0
Cops15.m6	-	-	1	5 606.9	0.7
Cops15.m2	Group (2H)	Family	5	5 608.2	2.0
Cops15.m4	Sire	Family	4	5 608.4	2.2
Cops15.m0	-	Family	2	5 608.6	2.4
Cops15.m1	Group (1H)	Family	4	5 610.7	4.5
Cops15.m5	Family	-	15	5 620.1	13.9
